# In House Rapid, Simple Multiple‐Locus Variable‐Number Tandem Repeat Analysis (MLVA): A Reliable Tool for *Enterobacter hormaechei* Genotyping

**DOI:** 10.1002/mbo3.70141

**Published:** 2025-11-09

**Authors:** Claire Guillermard, Audrey Baron, Philippe Bidet, Kevin La, Maud Gits‐Muselli, Céline Courroux, Célie Malaure, Laurent Dortet, André Birgy, Stéphane Bonacorsi

**Affiliations:** ^1^ Department of Microbiology Robert‐Debré Hospital, Assistance Publique – Hôpitaux de Paris Paris France; ^2^ University of Paris Cité, IAME, UMR 1137, INSERM Paris France; ^3^ Department of Microbiology Saint‐Antoine Hospital, Assistance Publique – Hôpitaux de Paris Paris France; ^4^ Department of Bacteriology‐Hygiene Bicêtre Hospital, Assistance Publique – Hôpitaux de Paris Le Kremlin‐Bicêtre France; ^5^ Faculty of Medicine, Team “Resist”, UMR1184, Immunology of Viral, Auto‐immune, Haematological and Bacterial diseases (IMVA‐HB), INSERM Paris‐Saclay University Le Kremlin‐Bicêtre France; ^6^ Associated French National Reference Center for Antibiotic Resistance: Carbapenemase‐producing Enterobacteriaceae Le Kremlin‐Bicêtre France

**Keywords:** *Enterobacter cloacae*, *Enterobacter hormaechei*, epidemic, genotyping method, MLST, MLVA, outbreak, typing method, WGS

## Abstract

*Enterobacter hormaechei*, a prominent species within the *Enterobacter cloacae* complex, is a significant cause of nosocomial infections and is frequently associated with multidrug resistance. Rapid genomic comparison helps guide timely infection control measures. This study aimed to develop a simple and rapid multiple‐locus variable‐number tandem‐repeat analysis (MLVA) protocol for epidemiological surveillance of *E. hormaechei*. Eight variable‐number tandem‐repeat (VNTR) regions were selected for amplification using multiplex PCR, followed by gel electrophoresis. The method's discriminatory power was evaluated on 46 unrelated strains from 22 French hospitals. Then, suspected related strains from three potential outbreaks, including ESBL‐ and/or NDM‐producing isolates were compared. An independent collection of 22 VIM‐producing strains was also analyzed. Whole‐genome sequencing (WGS) was used as the gold standard. Among 46 unrelated *E. hormaechei* strains, representing the five subspecies, MLVA and MLST showed similar discriminatory power (36 MLVA profiles vs. 33 STs, Hunter and Gaston diversity indices 0.9833 vs. 0.9824, respectively). Isolates with different ST typically had distinct MLVA profiles, except for three instances where different STs shared similar profiles. In STs represented by multiple strains, MLVA sometimes distinguished strains sharing the same ST. Among the three potential outbreak, epidemic strains exhibited unique MLVA profiles, with genetic distances of 0–11 SNPs using WGS, while unrelated isolates had different MLVA profiles, indicating this technique's potential as an effective screening tool for clonal groups. Similar results were observed for VIM‐producing *E. hormaechei*, with consistent MLVA profiles within the same STs. The MLVA protocol developed is a rapid, cost‐effective method for *E. hormaechei* epidemiological investigations that can quickly rule out unrelated strains. However, highly discriminatory techniques like WGS remain necessary when profiles are similar.

## Graphical abstract

We developed a new MLVA protocol targeting eight VNTR loci for the rapid and cost‐effective surveillance of *Enterobacter hormaechei*. Tested on diverse clinical isolates, it showed a discriminatory power comparable to MLST and was able to distinguish some strains within the same sequence type. This method offers a valuable first‐line tool for outbreak detection, although WGS remains necessary for high‐resolution analysis.

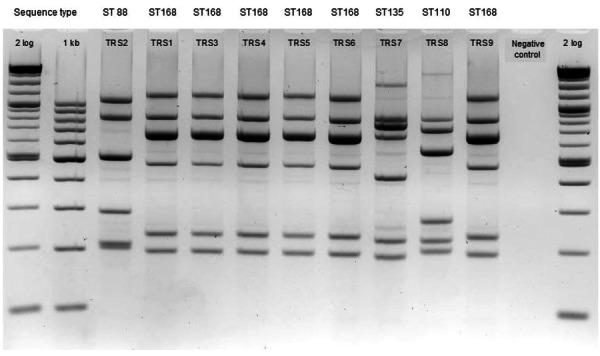

## 1 Introduction

The *Enterobacter cloacae* complex (ECC) comprises a group of widespread environmental organisms that are significant nosocomial pathogens involved in various infections (Mezzatesta et al. 2012). First, described by O'Hara et al. in 1989 through biochemical analysis of 23 strains, the ECC is still subject to taxonomic revisions due to its genetic diversity (O'Hara et al. 1989). Hoffmann and Roggenkamp (2003) initially identified 13 genetic clusters (I–XIII) within the complex using *hsp60* gene sequencing as the standard for species identification. This classification was later expanded to 18 clades (A–R) by Chavda et al. in 2016 and to 22 clades (A–V) by Sutton et al. in 2018 through whole‐genome sequencing (WGS) (Chavda et al. 2016).

Phenotypic methods have historically struggled to differentiate between ECC species, often misclassifying isolates as *E. cloacae* by MALDI‐TOF mass spectrometry. Recent improvements—including the development of a new MALDI‐TOF database update by Bruker Daltonics in 2023—have greatly enhanced species discrimination. These advances revealed that the *Enterobacter hormaechei* complex is the most frequent ECC member among clinical isolates (Chavda et al. 2016; Emeraud et al. 2022; Morand et al. 2009; Sutton et al. 2018). Five subspecies have been assigned to *E. hormaechei* named *oharae, hoffmannii, steigerwaltii, xiangfangensis,* and *hormaechei*, which are differentiated by molecular approaches (Chavda et al. 2016; Rezzoug et al. 2024).

Importantly, *E. hormaechei* stands out as a prominent nosocomial pathogen frequently associated with multidrug resistance (MDR) and is implicated in healthcare‐associated infections and outbreaks (8–13). Its ability to acquire and disseminate resistance determinants poses significant challenges in clinical settings. During outbreaks, it is essential to rapidly differentiate epidemic strains from unrelated ones to implement appropriate infection control measures and prevent further spread. Various genotyping tools have been applied to the ECC (14, 15), each with its own advantages and limitations. Pulsed‐field gel electrophoresis, the historical reference method, offers high discriminatory power but is lengthy and labor‐intensive. Although WGS is now the gold standard due to its high resolution, it remains time‐consuming, expensive for routine use and is not available in all laboratories

Therefore, there is a need for a simple and rapid alternative discriminatory method. We propose the application of multi‐locus variable‐number tandem repeat analysis (MLVA), which offers the benefits of PCR methods combined with high discriminatory power. In its standard form, MLVA requires capillary electrophoresis. However a rapid multiplex PCR‐based typing method with migration on agarose gel electrophoresis has already been established for organisms like *Escherichia coli*, *Klebsiella pneumoniae* and *Pseudomonas aeruginosa* with good performances (Caméléna et al. 2019; Legouge et al. 2023; Bidet et al. 2022). By exploiting the polymorphic nature of variable‐number tandem repeats (VNTR) dispersed across the bacterial chromosome, MLVA could provide a practical and effective tool for molecular epidemiology of *E. hormaechei* strains.

This study describes a method we developed that merges the simplicity, speed, and cost‐effectiveness of standard PCR with the high discriminatory power of MLVA, enabling the typing of *E. hormaechei* complex species. We compared our results with those from WGS to evaluate the method's effectiveness in epidemiological investigations.

## 2 Methods

### 2.1 Isolates

Sixty‐four non‐duplicate *E. hormaechei* complex isolates (63 from patients and one control strain “EEQ”) were included and sequenced in this study. First, 46 isolates from different clinical samples (urine, stool, vaginal swab, blood culture, bone biopsy) were randomly collected from unrelated adults and children presenting to the emergency department or hospitalization ward from 22 different French hospitals. They were used to evaluate the diversity of MLVA profiles obtained with our method. Second, three groups of potentially related strains from epidemic outbreaks were included: nine extended‐spectrum beta‐lactamase (ESBL)‐producing *E. hormaechei* complex from Armand Trousseau (TRS) hospital (Paris), four New Delhi Metallo‐beta‐lactamase (NDM)‐producing isolates and five ESBL and/or NDM‐producing isolates from children's stools hospitalized in the haematology or neonatal department of Robert Debré (RDB) hospital (Paris). These groups were used to assess the discriminating potential of the technique.

Third, as Verona Integron‐encoded Metallo‐beta‐lactamase (VIM) producing *E. hormaechei* complex strains are emerging in France, 22 isolates collected from different regions of France, which had been previously described and sequenced, were included (Emeraud et al. 2022). Some of these isolates are genetically related, while others are not. The characteristics of all these strains are described in Table SI.

### 2.2 Microbiology

All bacterial strains were initially identified using the updated MALDI‐TOF MS database (Bruker Daltonics, 2023). Antibiotic susceptibility testing was conducted using the disk diffusion method (Bio‐Rad) and results were interpreted using updated EUCAST breakpoint (effective January 1st, 2024) (EUCAST 2024).

### 2.3 MLVA Setup

The genome sequence of *E. hormaechei* FDAARGOS 1435 chromosome (GenBank accession no. NZ_CP077392.1) was used to identify potential tandem repeats with a tandem repeat finder tool (Benson 1999). Different VNTR loci were selected based on their variability of repeats among reference genome and their size range compatibility with other VNTRs. To confirm the presence of the selected VNTRs in other strains of *E. hormaechei*, we performed a BLAST analysis using 12 circular *E. hormaechei* genomes obtained from GenBank (accession numbers NZ_CP077392.1; NZ_CP033102.1; NZ_CP030347.1; NZ_CP090792.1; NZ_AP025923.1; NZ_CP118552.1; NZ_CP019889.1; NZ_CP110857.1; NZ_AP025842.1; NZ_CP058187.1; NZ_AP025799.1; NZ_CP049188.1).

We designed primers for each VNTR locus by aligning flanking sequences (*n* = 15) from *E. hormaechei* reference genomes using ClustalW (https://www.genome.jp/tools-bin/clustalw). All primers were selected to ensure that the size ranges allowed clear separation of bands and minimized the risk of overlap on electrophoresis gels. We retained the eight primer pairs that provided the highest level of discrimination (Table 1).

**Table 1:** Primers and variable‐number tandem repeats (VNTR) selected for the MLVA scheme.

VNTR	Primers	Sequence (5’‐3’)	Coordinates on *E. hormaechei* complete genome (GenBank accession no. NZ_CP077392.1)	Repeat unit size (bp)
H2	H2‐F	GTTCCCGCTGCGCCATGCGC	256 682 – 256 701	46
	H2‐R	AGCGTTGCCGCACCGGTAGTGGC	257 832 – 257 809	
H3	H3‐F	CCAACTGACCACCTCACCATTACG	179 683 – 179 706	18
	H3‐R	CGCATCAGAGTGGGTCTTCGCCTGGC	180 377 – 180 350	
H4	H4‐F	CGGAGTGCAGTGCGCTAGCGG	336 198 – 336 218	104
	H4‐R	GGATCCCCAGATGGGTGGTCTGCCCG	337 007 – 336 981	
H6	H6‐F	GGTTGCTGCTTTGGGCCACGG	1 671 326 – 1 671 347	15
	H6‐R	CCGTCGATCTGACCATCGCGC	1 672 394 – 1 672 374	
H8	H8‐F	CGAGCTGAACTATGTTTACGCG	1 813 002 – 1 813 023	58
	H8‐R	CCTGGGCTACGGTGGCGAACAGCCG	1 813 923 – 1 813 900	
H9	H9‐F	TTCGAATCCCCGCCTCACCGC	1 971 899 – 1 971 919	141
	H9‐R	AGATCAAACCGTCATACTGTGCG	1 973 267 – 1 973 245	
V7	V7‐F	TCCCATGCCGCGTATTTGCTGGC	2 844 535 – 2 844 554	48
	V7‐R	AAGCGACGGCAAAACCAGCGT	2 845 019 – 2 845 000	
V12	V12‐F	CTGACCATCGCGCAGAAAGA	4 421 535 – 4 421 554	24
	V12‐R	TGGAAAATCCCTGGCTTGCCGC	4 421 977 – 4 421 958	

### 2.4 MLVA Typing

To perform the MLVA typing analysis, bacterial strains were incubated overnight at 35°C on trypticase soy agar (TSA). Ultra‐rapid DNA extraction was carried out as previously described (Caméléna et al. 2019). Briefly, for this procedure, a 1 µL loop of bacteria was suspended in 1 mL of 0.9% NaCl. DNA extract was obtained by mixing one volume of bacterial suspension with two volumes of 100 mM NaOH.

MLVA analysis was performed through PCR amplification of the eight VNTRs using a set of primer mixes (Table 1). The eight VNTRs were amplified in a single‐tube multiplex PCR with a 25 µL reaction volume, utilizing a multiplex PCR kit (Qiagen, Hilden, Germany). The reaction mixture included 1 µL of DNA extract, 2.5 µL of primer mix (containing forward and reverse primers at 0.1 µM each), 2.5 µL of 5X Q solution, 12.5 µL of 2X Multiplex PCR Master Mix (Qiagen), and 6.5 µL of sterile water. The PCR conditions involved an initial denaturation step at 95°C for 15 min, followed by 30 cycles (denaturation at 94°C for 30 s, annealing at 55°C for 90 s, extension at 72°C for 90 s) with a final extension step at 72°C for 10 min. Gel electrophoresis was conducted on a 3% standard agarose gel stained with ethidium bromide. Amplicon sizes were estimated using the 2‐log DNA ladder as reference (BioLabs).

To compare fingerprints across different gels, we used BioNumerics 7.10 (Applied Maths, Sint‐Martens‐Latem, Belgium). MLVA patterns were analyzed using a tolerance parameter of 1% and an optimization parameter of 0.5%. Dendrograms were constructed using the unweighted pair group method with arithmetic mean and the pairwise Dice similarity coefficient. Profiles were considered identical if they exhibited the same banding pattern, corresponding to a similarity of over 95% on the dendrograms. The discriminatory power was quantified using the Hunter and Gaston (1988) diversity index.

### 2.5 WGS

WGS was then performed on all isolates. Genome assemblies were constructed using SPAdes software, and acquired antibiotic resistance genes were identified using ResFinder 3.0. Subspecies classification was determined based on WGS data analysis. Whole DNA was extracted using a MoBio kit (Qiagen). Libraries were prepared using DNA flex kit (Illumina, San Diego, CA, USA). Sequencing was performed on a NextSeq instrument for 2 × 150 cycles (Illumina), as described previously (Caméléna et al. 2019).

The SPAdes v3.15.4 (Using SPAdes 2020) assembler was used to construct assemblies. The quality of the sequencing data was estimated using standard metrics, including N50 given by QUAST v5.0.5 (Gurevich et al. 2013) and theoretical coverage. MLST (MLST v2.19.0) of *E. cloacae* was performed using profile database (2024‐06‐18) from https://pubmlst.org/. Based on MLST profile, a minimum spanning tree using grapetree v1.5.0 of achtman‐lab was performed (Zhou et al. 2018) (Figure S1).

**Figure 1:**
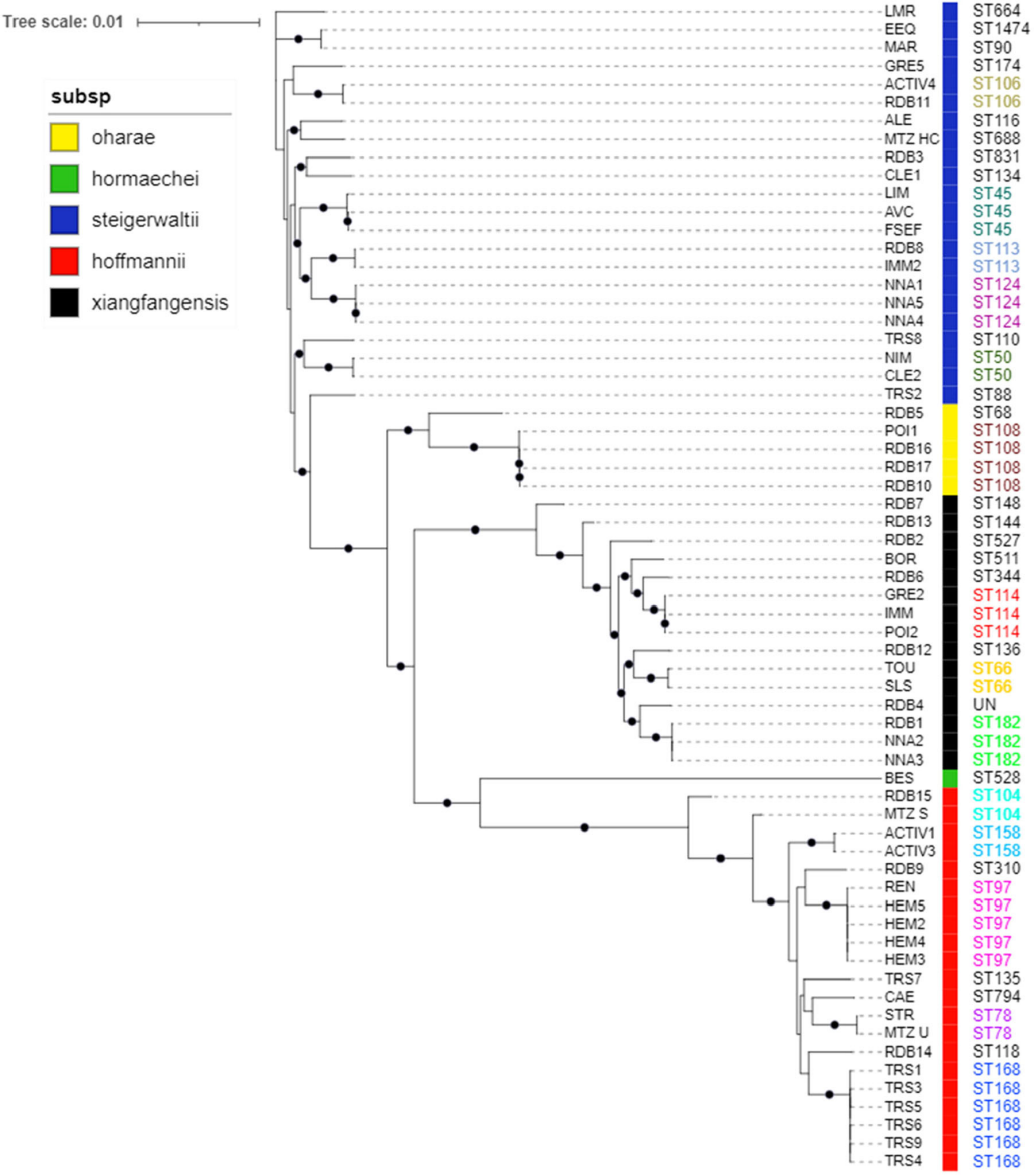
Phylogenetic tree of all 64 strains (unrelated strains and potential outbreak strains). Subspecies are indicated by colored squares (subsp). The nodes represent bootstrap values from a resampling analysis with 1000 iterations.

To visualize population structure, a phylogenetic tree was constructed usinq IQtree v1.6.9 (Nguyen et al. 2015) based on core genome alignment from panaroo v1.5.0 (Tonkin‐Hill et al. 2020) (Figure 1). Panaroo utilized annotations produced by prokka v1.14.5 (Seemann 2014). Then, a single‐nucleotide polymorphism (SNP) matrix was computed on core genome alignment using pairsnp v0.0.1 (https://github.com/gtonkinhill/pairsnp) (Table SII).

Subspecies were identified by comparing all sequences in fasta format to the closest reference genome described by Valentina Donà et al (Emergence 2024). using MASH v2.3 (Ondov et al. 2016).

## 3 Results

### 3.1 MLVA Setup

Tandem repeat finder tool identified 20 different VNTRs loci on *E. hormaechei* FDAARGOS 1435 chromosome. We then retained eight VNTRs that were present in the 12 different genomes of *E. hormaechei* obtained from GenBank. Primers were then designed to amplify VNTR regions and to produce different size ranges allowing a correct visualization by maximizing the discrimination of the eight bands on gel electrophoresis after amplification. Details of the primers are provided in Table 1. To assess the presence of discriminatory amplicons on different isolates, we mimicked an electrophoresis gel using an *in‐silico* approach to observe the presence of discriminating bands and the different MLVA profiles of the 12 circular *E. hormaechei* genomes (Figure S2). Among the 12 isolates, we obtained 10 different patterns with 7 or 8 bands each, indicating a strong potential for the discriminatory power of these eight VNTRs. To confirm that VNTRs loci were not clustered in the genome, we determined their positions in the genome of *E. hormaechei* strain FDAARGOS 1435 chromosome (Figure S3).

### 3.2 MLVA and WGS Comparison

MLVA was performed successfully on all 64 *E. hormaechei* isolates. WGS was also achieved to determine the sequence type (ST) *in silico* and to conduct genomes comparison through SNP analysis. The median N50 was 157,185 and the coverage was 34X. Details of standard metrics of WGS are provided in Table SI. Raw reads have been deposited in GenBank under BioProject ID: PRJNA1150570.

Genetic diversity and relationships between strains of our collection, including those involved in epidemics, were analyzed through SNP analysis (Table SII) and are represented on Figure 1. This allowed us to divide the 64 sequenced strains into five subgroups, each corresponding to a different subspecies of *E. hormaechei* complex as described in Section 2. Three subspecies appear to be predominant: *E. steigerwaltii*, *E. hoffmannii*, and *E. xiangfangensis*.

### 3.3 MLVA Performed on Unrelated Isolates

Forty‐six unrelated isolates from different French hospitals were used to assess the discriminatory power of our MLVA method on *E. hormaechei* complex strains. The calculations of the Hunter and Gaston diversity index yielded values of 0.9833 for MLVA typing and 0.9824 for MLST, showing very similar discriminatory powers for MLVA and MLST.

**Figure 2:**
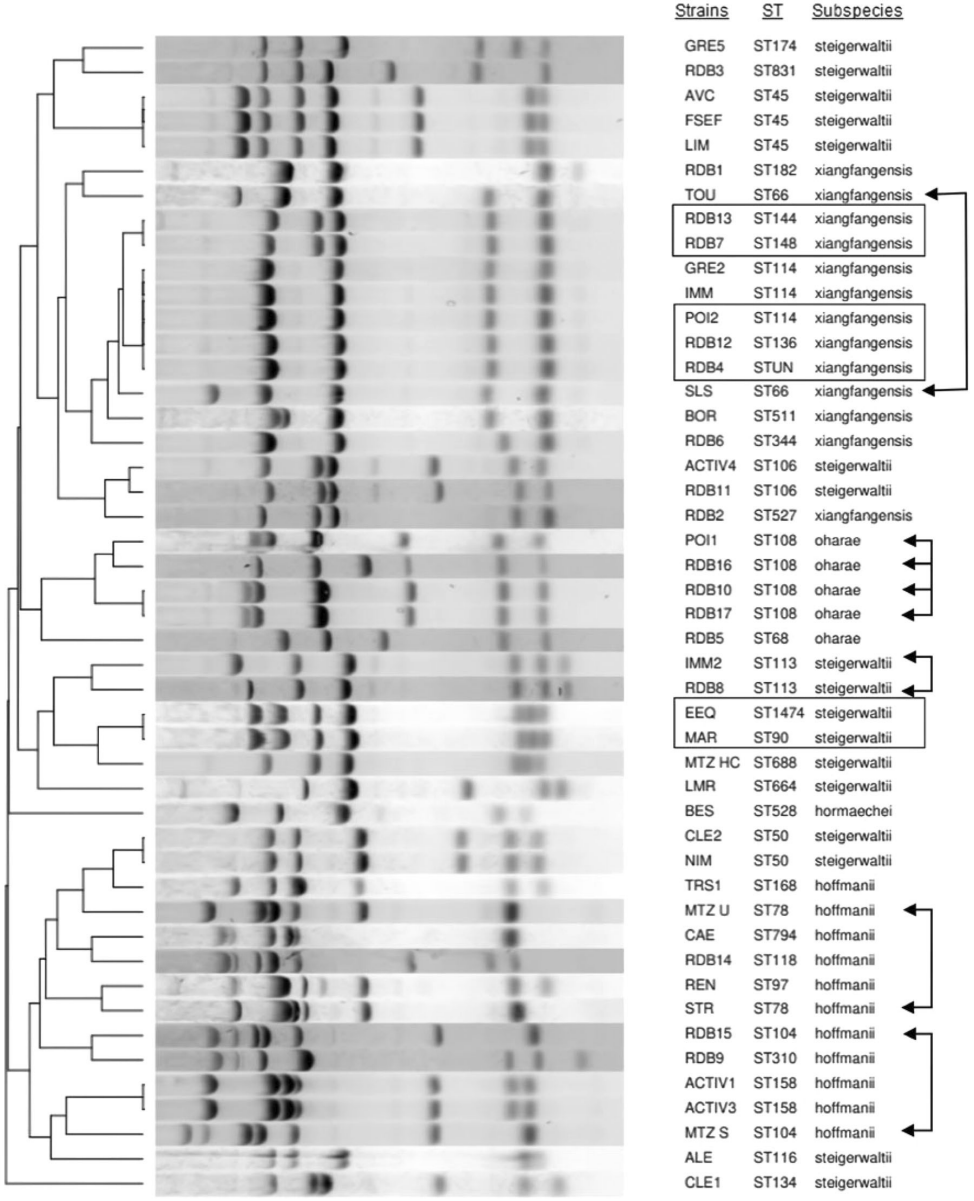
Dendogram of the MLVA results of unrelated *Enterobacter hormaechei* complex strains. The dendogram was constructed using Bionumerics software with the unweight pair group method using average linkages and the Dice algorithm, based on the band profiles from the electrophoresis gel. MLVA differentiates strains within the same ST (arrows). MLVA profiles were not distinguishable between ST144 and ST148, between ST136, ST114, and STUN, and between ST90 and ST1474 (squares). Squares indicate the same MLVA profile across different STs; arrows indicate the same ST with different MLVA profiles. ST, sequence type; STUN, unknown ST.

For 21 STs, a distinct MLVA profile was obtained and for some (*n* = 5) MLVA was even able to distinguish between strains in the same ST (represented by arrows in Figure 2).

However, MLVA profiles were not distinguishable between ST144 and ST148, between ST136, ST114, and STUN, and between ST90 and ST1474 (indicated by squares in Figure 2). Core‐genome SNP analysis showed approximately 20,000 SNP differences among some strains that shared identical MLVA profiles (e.g., ST144 vs. ST148; ST136, vs. ST114, vs. STUN). In contrast, EEQ (ST90) and MAR (ST1474) also shared the same MLVA profile but differed by 269 SNPs only—fewer than certain strains within the same ST complex, differing by only one allele.

### 3.4 Analysis of Potential Outbreaks

Three potential outbreaks of *E. hormaechei* complex infections were identified in three departments of two Parisian hospitals. A potential outbreak was defined as strains isolated in the same department within a limited time frame. These isolates were used to challenge our MLVA method.

**Figure 3:**
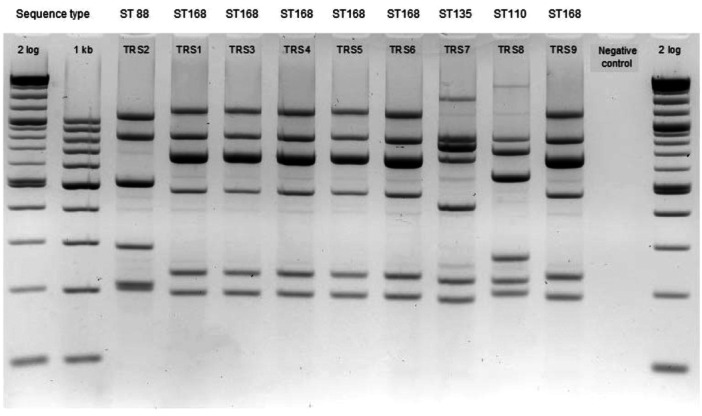
MLVA results for nine ESBL‐producing *E. hormaechei* complex isolates in the neonatal department of TRS hospital between June and October 2023. 2 log: 2‐log molecular weight; 1 kb: 1000 base‐pairs molecular weight.

In the first hospital, in neonatology department, nine ESBL or NDM‐producing strains were isolated between June and October 2023. Six strains belonged to *E. hofmannii* ST168 and shared the same MLVA profile. The three remaining strains had unique MLVA profiles corresponding to *E. steigerwaltii* ST88 (TRS2), *E. steigerwaltii* ST110 (TRS8), and *E. hofmannii* ST135 (TRS7) (Figure 3). The six *E. hofmannii* ST168 strains differed by 0 to 7 SNPs (Table SII) and isolates TRS 2, 7, and 8 showed a difference of 23,000 to 119,000 SNPs.

**Figure 4:**
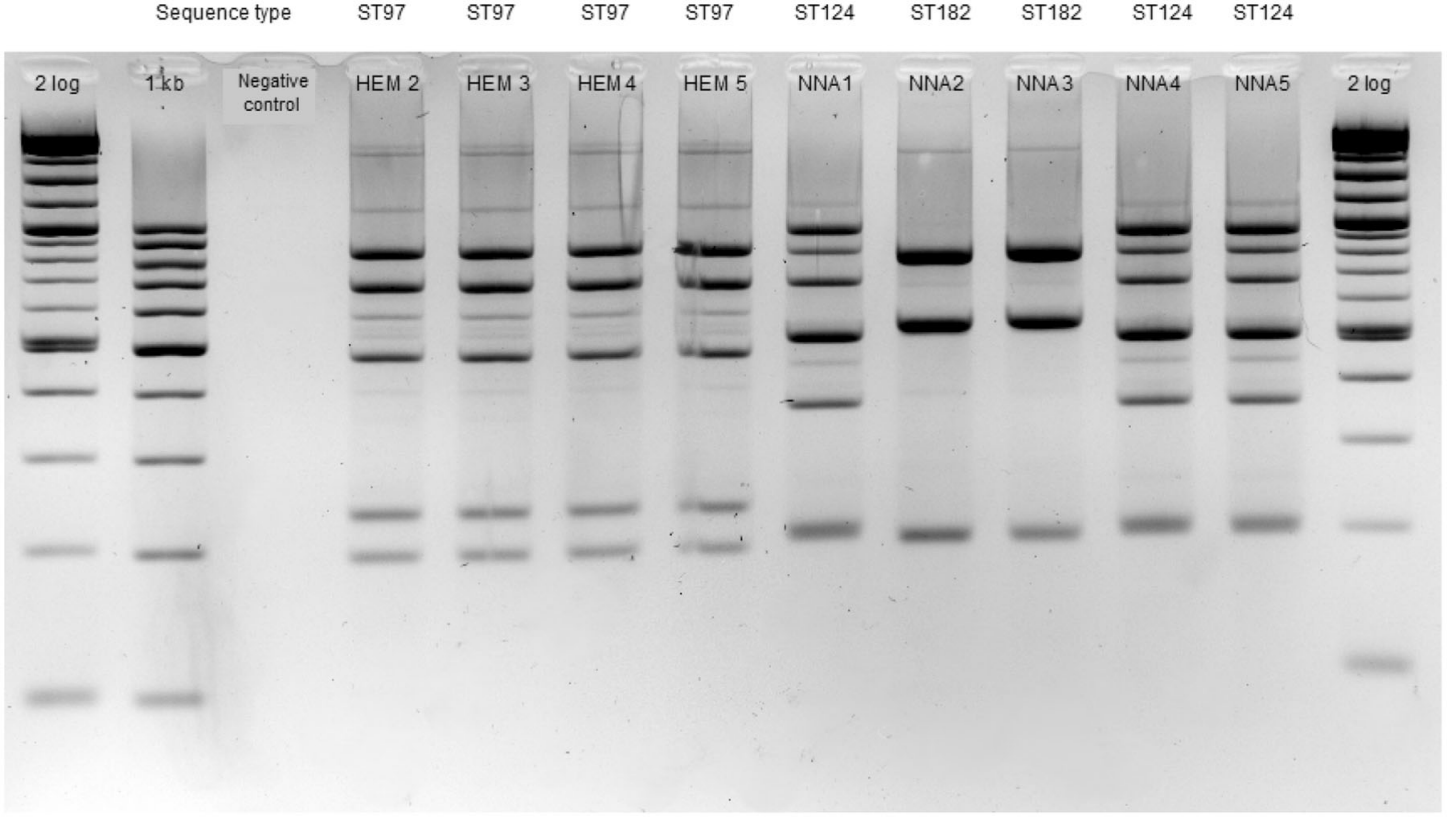
MLVA results for five ESBL‐producing *E. hormaechei* complex in neonatal (NNA1–NNA5) and four ESBL‐ and NDM‐producing *E. hormaechei* (HEM2 to HEM5) in haematology unit in RDB hospital. 2 log: 2‐log molecular weight; 1 kb: 1000 base‐pairs molecular weight.

In the second hospital, within the neonatology department of RDB hospital, five ESBL‐producing strains were identified between January 2023 and January 2024. In the haematology department, four ESBL‐ and NDM‐producing isolates were identified between March 2022 and June 2023. In the neonatology unit, two distinct MLVA profiles were observed with each profile corresponding to a specific ST (ST182 and ST124) (Figure 4). SNPs analysis showed that NNA2 and NNA3 were separated by 59 SNPs while NNA1, NNA4, and NNA5 were separated by 0 to 2 SNPs (Table SII). Strains from patients hospitalized in the haematology unit had identical MLVA profiles and belonged to the same *E. hofmannii* ST97 (Figure 4). SNP analysis revealed a difference of 0 to 11 SNPs among the four HEM strains.

### 3.5 MLVA Performed on *Carbapenemase* Vim‐Producing *E. hormaechei* Complex From France

Finally, to evaluate our MLVA method on an independent collection of strains, already sequenced and described, we analyzed 22 VIM carbapenemase‐producing *E. hormaechei* complex isolates collected from different regions of France (Emeraud et al. 2022).

From 2015 to 2018, the French National Reference Center (FNRC) for antimicrobial resistance observed a significant increase of the number of VIM‐producing ECC isolates raising concerns due to the therapeutic challenges they pose in case of infection (Emeraud et al. 2022).

**Figure 5:**
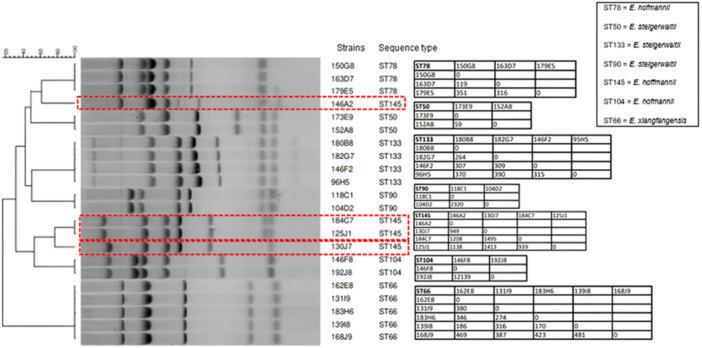
Dendogram of the MLVA results for VIM‐producing *Enterobacter hormaechei* complex strains, along with corresponding SNP pairwise comparisons from Emeraud et al. (2022). The number of SNPs between two strains is indicated in the matrix. The dendrogram was constructed using Bionumerics software with the unweighted pair group method, employing average linkages and the Dice algorithm based on the band profiles from the electrophoresis gel. Each ST had a unique MLVA profile except for *E. hofmannii* ST145 (outlined with a red dashed box).

Among the 22 VIM‐producing *E. hormaechei* complex strains representative of those collected by the FNRC, seven different STs were observed, and nine distinct MLVA profiles were identified. Each ST had a unique MLVA profile except for *E. hofmannii* ST145. Among the four *E. hofmannii* ST145 strains, three different profiles were detected (Figure 5). All *E. hofmannii* ST145 strains were differentiated by 939 to 1495 SNPs.

### 3.6 Repeatability and Stability of MLVA Typing

Two strains with different STs were subcultured daily for 14 days. MLVA patterns were assessed on days 0 to 7 and 14 with no difference observed, indicating the stability of the VNTR and the method even after numerous subcultures (Figure S4). This demonstrates that an MLVA profile determined at a given time can be reliably used for subsequent analyses.

## 4 Discussion

In this study, we developed a rapid and simple technical approach based on MLVA to type strains of *E. hormaechei*, a major specie belonging to ECC and involved in hospital acquired infection. In France, the ECC is classified as the third most common bacterial pathogen, following *E. coli* and *K. pneumoniae* known for harboring carbapenemase and ESBL encoding genes (Jousset 2021; Chabaud 2021).

First, we found that each strains forming a single ST has its own MLVA type with few exceptions. MLVA provided indeed finer resolution for certain STs, distinguishing strains that shared the same ST. However, some strains with different ST with a difference of nearly 20,000 SNPs had identical MLVA profiles—especially in *E. xiangfangensis* subspecies—revealing limitations in resolving genetically distant isolates when fewer amplicons are generated. Notably, these strains belong to the *E. xiangfangensis* subspecies, for which MLVA produced only 4–6 bands, in contrast to other subspecies that typically generate 6–7 bands. This suggests that MLVA may have a more limited discriminatory power for this subspecies.

We also assessed three potential epidemics of MDR strains from two Parisian's hospitals. Among the three studied outbreaks, MLVA successfully identified several unrelated strains in two departments, highlighting the main strength of this technique. This finding was subsequently corroborated by SNP analysis. A difference of approximately fewer than 20 SNPs being more supportive of clonality and a common source for these MDR strains. In contrast, a large number of SNP differences suggests that the strains are unrelated to each other and therefore do not belong to the same outbreak. Epidemic strains exhibited unique MLVA profiles, suggesting that this technique could be an effective screening tool for identifying clonal groups. However, WGS analysis should be performed when similar MLVA profiles are found to provide deeper insights for the epidemiological investigation.

Finally, we evaluated the performance of our MLVA method on a collection of 22 strains VIM‐carbapenemase‐producing *E. hormaechei*. One unique MLVA profile was observed by ST except for *E. hoffmannii* ST145 strains which were differentiated by ≥ 900 SNPs suggesting they are divergent strains.

Nonetheless, the high diversity of STs observed in *E. hormaechei* complex, including ESBL‐ and carbapenemase‐producing isolates (Emeraud et al. 2022; Rezzoug et al. 2024; Emergence 2024; Dortet et al. 2017; Peirano et al. 2018; Knecht et al. 2022) suggests a wide diversity of circulating strains. In the event of an outbreak, similar STs are expected. Therefore, the MLVA technique could be useful for quickly ruling out strains that are not involved in the outbreak.

To conclude, we propose a strategy for the epidemiological investigation of grouped cases of *E. hormaechei* complex that involves the sequential use of two molecular techniques. First, a simplified MLVA method was developed that requires only basic molecular biology equipment (a thermal cycler and agarose gel electrophoresis). This method is useful, fast (∼4 h), inexpensive (less than 4 USD), reliable and could be easily integrated into routine hospital workflows. Therefore, it allows rapid exclusion of most sporadic isolates from further analyses within a single day. Indeed, two different MLVA profiles clearly indicate that strains are unrelated, which is sufficient to rule out strains from potential outbreaks. This approach is particularly valuable for *E. hormaechei* complex, which are responsible for nosocomial outbreaks, especially those involving MDR strains. Second, WGS can be used to precisely categorize isolates that are indistinguishable by MLVA as potentially related or not. Due to the high clonality of some strains associated with nosocomial infections, basic typing methods with moderate discriminatory power are not sufficient, making WGS essential to draw firm conclusions. Thus, simplified MLVA facilitates rapid screening, allowing laboratories to focus their efforts on potentially related strains. Moreover, this method represents also an easy tool for differentiating ECC colonies when analyzing microbiota in a research laboratory context.

## Author Contributions

**Claire Guillermard:** writing – original draft, methodology, writing – review and editing, software. **Audrey Baron:** conceptualization, investigation, writing – review and editing, software. **Philippe Bidet:** conceptualization, methodology, validation, software. **Kevin La:** software. **Maud Gits‐Muselli:** investigation, methodology, writing – review and editing. **Céline Courroux:** methodology. **Célie Malaure:** resources. **Laurent Dortet:** methodology, resources, writing – review and editing. **André Birgy:** supervision, writing – review and editing, methodology. **Stéphane Bonacorsi:** conceptualization, investigation, methodology, writing – review and editing, supervision.

## Ethics Statement

The authors have nothing to report.

## Conflicts of Interest

The authors declare no conflicts of interest.

## Supporting information


**Supplemental Figure 1**: Minimum spanning tree of unrelated strains and representative strains from each sequence type (ST) within the VIM collection, EnteroMSTree ‐ GrapeTree (Zhou et al. 2018). STs with identical MLVA profiles are identified by arrows. Numbers between each ST indicate allelic difference. Subspecies are depicted as follows: dark blue for *steigerwalti*i, light blue for *hoffmannii*, orange for *xiangfangensis*, light orange for *oharae*, and green for *hormaechei*. **Supplemental Figure 2**: In silico gel of MLVA using eight VNTRs amplification of 12 *E. hormaechei* complex strains from https://www.ncbi.nlm.nih.gov/datasets/genome/. Each strain in lines 2 to 13 is named by its GenBanq accession number. 2 log WM: 2‐log weight molecular. **Supplemental Figure 3**: Sequence DNA of *E. hormaechei* complete genome (GenBank accession no. NZ_CP077392.1) and position of the 8 VNTRs, SnapGen®. **Supplemental Figure 4**: MLVA results for three strains after 14 days of subculturing. D0, day 0; D7, day 7; D14, day 14; 2 log: 2‐log molecular weight; 1 kb: 1000 base‐pairs molecular weight.

**Supplemental Table 1:** Characteristics of 64 *E. hormaechei* strains sequenced in this study.

**Supplemental Table 2:** SNPs matrix of the 64 *E. hormaechei* complex strains sequenced.

## Data Availability

The genomic data are presented in this study can be found in online repositories on the NCBI website under BioProject ID: PRJNA1150570.

## References

[mbo370141-bib-0001] Benson, G. 1999. “Tandem Repeats Finder: A Program to Analyze DNA Sequences.” Nucleic Acids Research 27: 573–580. 10.1093/nar/27.2.573. 9862982 PMC148217

[mbo370141-bib-0002] Bidet, P. , A. Birgy , B. Brethon , et al. 2022. “Epidemiological Investigation of *Pseudomonas aeruginosa* Isolates Including Multidrug‐Resistant Serogroup O12 Isolates, by Use of a Rapid and Simplified Multiple‐Locus Variable‐Number of Tandem Repeats Analysis and Whole Genome Sequencing.” Journal of Hospital Infection 130: 56–62. 10.1016/j.jhin.2022.09.012. 36181986

[mbo370141-bib-0003] Caméléna, F. , A. Birgy , Y. Smail , et al. 2019. “Rapid and Simple Universal *Escherichia coli* Genotyping Method Based on Multiple‐Locus Variable‐Number Tandem‐Repeat Analysis Using Single‐Tube Multiplex PCR and Standard Gel Electrophoresis.” Applied and Environmental Microbiology 85: e02812‐18. 10.1128/AEM.02812-18. 30610078 PMC6414366

[mbo370141-bib-0004] Chabaud, A. 2021. “Consommation d'antibiotiques et résistances bactériennes en établissement de santé. Données Spares 2020/Antibiotic Use and Antibiotic Resistance in French Healthcare Facilities in 2020: Data From the National SPARES Network.”

[mbo370141-bib-0005] Chavda, K. D. , L. Chen , D. E. Fouts , et al. 2016. “Comprehensive Genome Analysis of Carbapenemase‐Producing *Enterobacter* spp.: New Insights Into Phylogeny, Population Structure, and Resistance Mechanisms.” mBio 7: e02093‐16. 10.1128/mBio.02093-16. 27965456 PMC5156309

[mbo370141-bib-0006] Dortet, L. , G. Cuzon , V. Ponties , and P. Nordmann . 2017. “Trends in Carbapenemase‐Producing Enterobacteriaceae, France, 2012 to 2014.” Eurosurveillance 22: 1–9. 10.2807/1560-7917.ES.2017.22.6.30461. PMC5316908 28205502

[mbo370141-bib-0007] Emeraud, C. , C. Petit , L. Gauthier , R. A. Bonnin , T. Naas , and L. Dortet . 2022. “Emergence of VIM‐Producing *Enterobacter cloacae* Complex in France Between 2015 and 2018.” Journal of Antimicrobial Chemotherapy 77: 944–951. 10.1093/jac/dkab471. 35045171

[mbo370141-bib-0008] “Emergence of OXA‐48‐Producing *Enterobacter hormaechei* in a Swiss Companion Animal Clinic and Their Genetic Relationship to Clinical Human Isolates ‐ PubMed n.d.” Accessed July 23, 2024. https://pubmed.ncbi.nlm.nih.gov/37923369/. 10.1093/jac/dkad337 37923369

[mbo370141-bib-0009] EUCAST . 2024. “European Committee on Antimicrobial Susceptibility Testing. EUCAST Breakpoint Tables.”

[mbo370141-bib-0010] Gurevich, A. , V. Saveliev , N. Vyahhi , and G. Tesler . 2013. “QUAST: Quality Assessment Tool for Genome Assemblies.” Bioinformatics 29: 1072–1075. 10.1093/bioinformatics/btt086. 23422339 PMC3624806

[mbo370141-bib-0011] Hoffmann, H. , and A. Roggenkamp . 2003. “Population Genetics of the Nomenspecies *Enterobacter cloacae* .” Applied and Environmental Microbiology 69: 5306–5318. 10.1128/AEM.69.9.5306-5318.2003. 12957918 PMC194928

[mbo370141-bib-0012] Hunter, P. R. , and M. A. Gaston . 1988. “Numerical Index of the Discriminatory Ability of Typing Systems: An Application of Simpson's Index of Diversity.” Journal of Clinical Microbiology 26: 2465–2466. 10.1128/jcm.26.11.2465-2466.1988. 3069867 PMC266921

[mbo370141-bib-0013] Jousset, A. B. 2021. “Caractéristiques et évolution des souches d'entérobactéries productrices de carbapénémases (EPC) isolées en France, 2012‐2020/Characteristics and evolution of carbapenemase‐producing Enterobacterales in France, 2012‐2020.”

[mbo370141-bib-0014] Knecht, C. A. , N. García Allende , V. E. Álvarez , et al. 2022. “New Sequence Type of an *Enterobacter cloacae* Complex Strain With the Potential to Become a High‐Risk Clone.” Journal of Global Antimicrobial Resistance 31: 162–164. 10.1016/j.jgar.2022.08.015. 36049730

[mbo370141-bib-0015] Legouge, C. , P. Bidet , M. Gits‐Muselli , et al. 2023. “Rapid, Simple Multi‐Locus Variable Number Tandem Repeat Analysis: A Reliable Tool for *Klebsiella pneumoniae* Outbreak Screening.” Journal of Hospital Infection 141: 41–48. 10.1016/j.jhin.2023.08.010. 37634603

[mbo370141-bib-0016] Mezzatesta, M. L. , F. Gona , and S. Stefani . 2012. “ *Enterobacter cloacae* Complex: Clinical Impact and Emerging Antibiotic Resistance.” Future Microbiology 7: 887–902. 10.2217/fmb.12.61. 22827309

[mbo370141-bib-0017] Morand, P. C. , A. Billoet , M. Rottman , et al. 2009. “Specific Distribution Within the *Enterobacter cloacae* Complex of Strains Isolated From Infected Orthopedic Implants.” Journal of Clinical Microbiology 47: 2489–2495. 10.1128/JCM.00290-09. 19515837 PMC2725656

[mbo370141-bib-0018] Nguyen, L.‐T. , H. A. Schmidt , A. Von Haeseler , and B. Q. Minh . 2015. “IQ‐TREE: A Fast and Effective Stochastic Algorithm for Estimating Maximum‐Likelihood Phylogenies.” Molecular Biology and Evolution 32: 268–274. 10.1093/molbev/msu300. 25371430 PMC4271533

[mbo370141-bib-0019] O'Hara, C. M. , A. G. Steigerwalt , B. C. Hill , J. J. Farmer , G. R. Fanning , and D. J. Brenner . 1989. “ *Enterobacter hormaechei*, a New Species of the Family Enterobacteriaceae Formerly Known as Enteric Group 75.” Journal of Clinical Microbiology 27: 2046–2049. 10.1128/jcm.27.9.2046-2049.1989. 2778068 PMC267735

[mbo370141-bib-0020] Ondov, B. D. , T. J. Treangen , P. Melsted , et al. 2016. “Mash: Fast Genome and Metagenome Distance Estimation Using Minhash.” Genome Biology 17: 132. 10.1186/s13059-016-0997-x. 27323842 PMC4915045

[mbo370141-bib-0021] Peirano, G. , Y. Matsumura , M. D. Adams , et al. 2018. “Genomic Epidemiology of Global Carbapenemase‐Producing *Enterobacter* spp., 2008–2014.” Emerging Infectious Diseases 24: 1010–1019. 10.3201/eid2406.171648. 29774858 PMC6004858

[mbo370141-bib-0022] Rezzoug, I. , C. Emeraud , C. Rodriguez , J.‐M. Pawlotsky , R. A. Bonnin , and L. Dortet . 2024. “Regional Dissemination of NDM‐1 Producing *Enterobacter hormaechei* ST1740, With a Subset of Strains Co‐Producing VIM‐4 or IMP‐13, France, 2019 to 2022.” Eurosurveillance 29: 1–8. 10.2807/1560-7917.ES.2024.29.11.2300521. PMC10941310 38487887

[mbo370141-bib-0023] Seemann, T. 2014. “Prokka: Rapid Prokaryotic Genome Annotation.” Bioinformatics 30: 2068–2069. 10.1093/bioinformatics/btu153. 24642063

[mbo370141-bib-0024] Sutton, G. G. , L. M. Brinkac , T. H. Clarke , and D. E. Fouts . 2018. “ *Enterobacterhormaechei* subsp. *hoffmannii* subsp. nov., *Enterobacter hormaechei* subsp. *xiangfangensis* comb. nov., *Enterobacter roggenkampii* sp. nov., and *Enterobacter muelleri* Is a Later Heterotypic Synonym of *Enterobacter asburiae* based on Computational Analysis of Sequenced *Enterobacter* Genomes.” F1000Research 7: 521. 10.12688/f1000research.14566.2. 30430006 PMC6097438

[mbo370141-bib-0025] Tonkin‐Hill, G. , N. MacAlasdair , C. Ruis , et al. 2020. “Producing Polished Prokaryotic Pangenomes With the Panaroo Pipeline.” Genome Biology 21: 180. 10.1186/s13059-020-02090-4. 32698896 PMC7376924

[mbo370141-bib-0026] Using SPAdes . 2020. “De Novo Assembler ‐ Prjibelski ‐ 2020 ‐ Current Protocols in Bioinformatics ‐ Wiley Online Library n.d.” Accessed July 23, 2024. https://currentprotocols.onlinelibrary.wiley.com/doi/10.1002/cpbi.102. 10.1002/cpbi.102 32559359

[mbo370141-bib-0027] Zhou, Z. , N.‐F. Alikhan , M. J. Sergeant , et al. 2018. “Grapetree: Visualization of Core Genomic Relationships Among 100,000 Bacterial Pathogens.” Genome Research 28: 1395–1404. 10.1101/gr.232397.117. 30049790 PMC6120633

