# Supplementary material for: In House Rapid, Simple Multiple‐Locus Variable‐Number Tandem Repeat Analysis (MLVA): A Reliable Tool for Enterobacter hormaechei Genotyping
Source: Microbiologyopen. 2025 Nov 9;14(6):e70141. doi: 10.1002/mbo3.70141 (PMC12597776; doi:10.1002/mbo3.70141)

**Supplemental figures**

Supplemental Figure 1


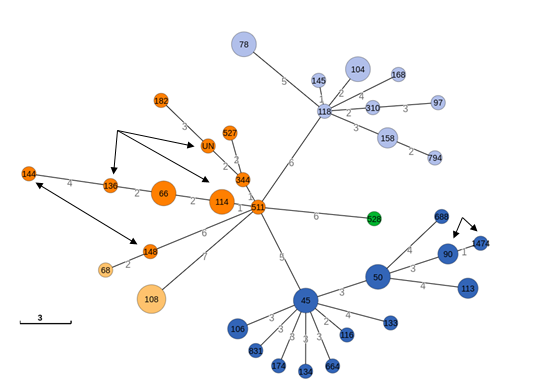


Supplemental Figure 2


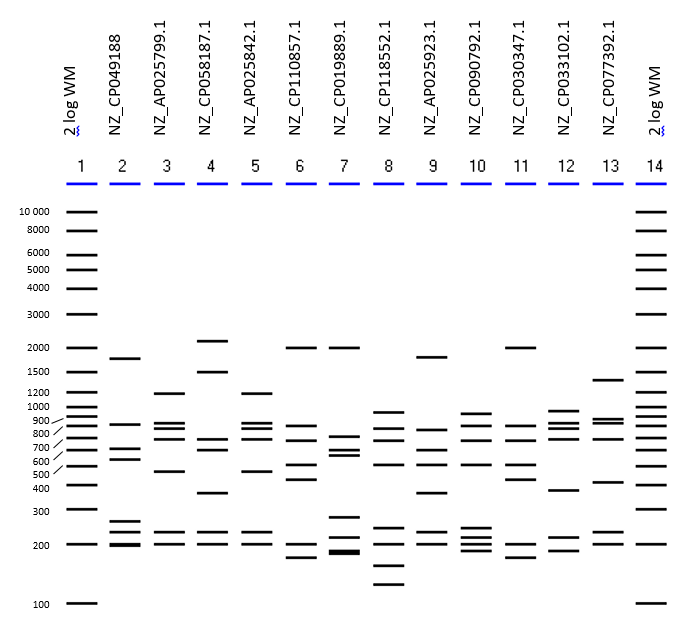


Supplemental Figure 3


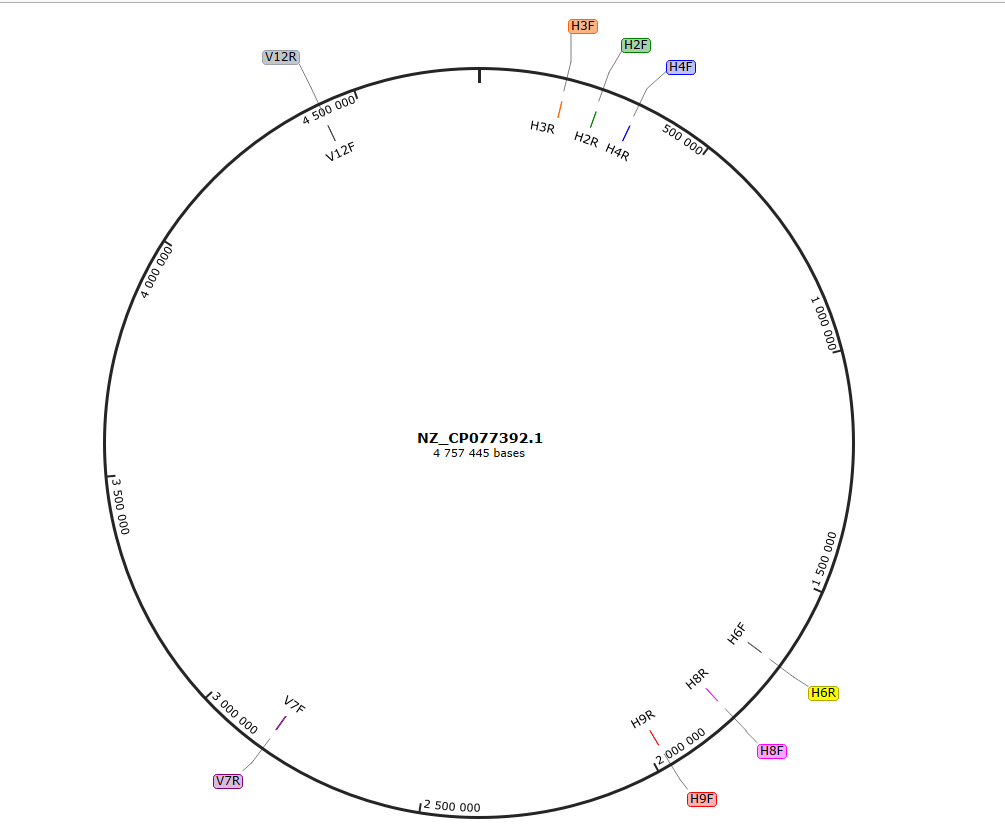


Supplemental Figure 4


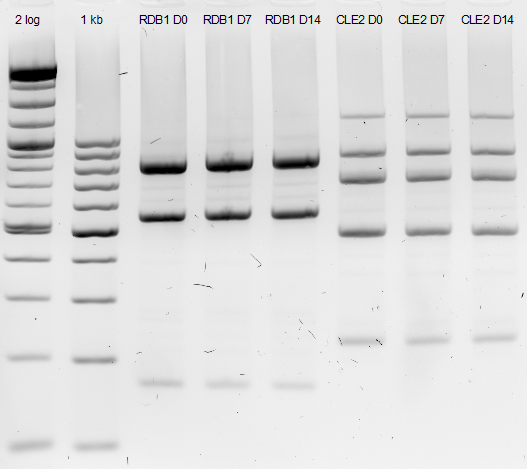

Supplement: Supplementary file 1 — Supplemental Figure 1: Minimum spanning tree of unrelated strains and representative strains from each sequence type (ST) within the VIM collection, EnteroMSTree ‐ GrapeTree (Zhou et al. 2018). STs with identical MLVA profiles are identified by arrows. Numbers between each ST indicate allelic difference. Subspecies are depicted as follows: dark blue for steigerwaltii, light blue for hoffmannii, orange for xiangfangensis, light orange for oharae, and green for hormaechei. Supplemental Figure 2: In silico gel of MLVA using eight VNTRs amplification of 12 E. hormaechei complex strains from https://www.ncbi.nlm.nih.gov/datasets/genome/. Each strain in lines 2 to 13 is named by its GenBanq accession number. 2 log WM: 2‐log weight molecular. Supplemental Figure 3: Sequence DNA of E. hormaechei complete genome (GenBank accession no. NZ_CP077392.1) and position of the 8 VNTRs, SnapGen®. Supplemental Figure 4: MLVA results for three strains after 14 days of subculturing. D0, day 0; D7, day 7; D14, day 14; 2 log: 2‐log molecular weight; 1 kb: 1000 base‐pairs molecular weight. [file MBO3-14-e70141-s003.docx]
