# Supplementary material for: In House Rapid, Simple Multiple‐Locus Variable‐Number Tandem Repeat Analysis (MLVA): A Reliable Tool for Enterobacter hormaechei Genotyping
Source: Microbiologyopen. 2025 Nov 9;14(6):e70141. doi: 10.1002/mbo3.70141 (PMC12597776; doi:10.1002/mbo3.70141)
Supplement: Supplementary file 2 — Supplemental Table 1: Characteristics of 64 E. hormaechei strains sequenced in this study. [file MBO3-14-e70141-s002.docx]

Supplemental Table 1: Characteristics of 64 *E. hormaechei* strains sequenced in this study.

| Sample's name | Sample's date | Sample’s origin  City (hospital) | Clinical sample's type | Resistance phenotype | Number of contigs | N50 | Coverage (x) | Subspecies | ST |
| --- | --- | --- | --- | --- | --- | --- | --- | --- | --- |
| RDB10 | 2019 | Paris (RDB) | Stools | ESBL | 326 | 152352 | 33.29 | *E. oharae* | 108 |
| RDB7 | 2019 | Paris (RDB) | Stools | ESBL | 210 | 183504 | 36.54 | *E. xiangfangensis* | 148 |
| EEQ | 2023 | EEQ | Quality evaluation | ESBL | 182 | 268569 | 39.95 | *E. steigerwaltii* | 1474 |
| RDB17 | 2020 | Paris (RDB) | Stools | ESBL | 330 | 119763 | 28.44 | *E. oharae* | 108 |
| RDB2 | 2020 | Paris (RDB) | Stools | ESBL | 229 | 180509 | 25.69 | *E. xiangfangensis* | 527 |
| BOR | 2023 | Bordeaux | Bones | WT | 99 | 235995 | 20.10 | *E. xiangfangensis* | 511 |
| TOU | 2023 | Tours | Blood culture | ESBL | 213 | 210436 | 37.92 | *E. xiangfangensis* | 66 |
| SLS | 2023 | Paris (SLS) | Blood culture | CPE | 365 | 152383 | 29.13 | *E. xiangfangensis* | 66 |
| STR | 2023 | Strasbourg | Blood culture | HCASE | 248 | 150622 | 33.63 | *E. hoffmannii* | 78 |
| MAR | 2023 | Marseille | Blood culture | CPE | 261 | 122310 | 21.08 | *E. steigerwaltii* | 90 |
| FSEF | 2021 | Paris (FSEF clinic) | Blood culture | CPE | 137 | 405560 | 36.13 | *E. steigerwaltii* | 45 |
| GRE2 | 2023 | Grenoble | Blood culture | ESBL | 207 | 225704 | 22.80 | *E. xiangfangensis* | 114 |
| IMM | 2021 | Paris (IMM) | Blood culture | HCASE | 233 | 239688 | 26.55 | *E. xiangfangensis* | 114 |
| MTZ HC | 2023 | Metz | Blood culture | WT | 127 | 233824 | 35.62 | *E. steigerwaltii* | 688 |
| LMR | 2023 | Paris (LMR) | Blood culture | HCASE | 283 | 155169 | 29.77 | *E. steigerwaltii* | 664 |
| RDB14 | 2018 | Paris (RDB) | Blood culture | WT | 285 | 145210 | 54.92 | *E. hoffmannii* | 118 |
| GRE5 | 2023 | Grenoble | Bones | WT | 104 | 293080 | 22.43 | *E. steigerwaltii* | 174 |
| CAE | 2023 | Caen | Horse wound | ESBL | 257 | 158777 | 37.77 | *E. hoffmannii* | 794 |
| RDB8 | 2023 | Paris (RDB) | Superficial pus | HCASE | 129 | 222617 | 37.43 | *E. steigerwaltii* | 113 |
| RDB5 | 2023 | Paris (RDB) | Vaginal swab | WT | 133 | 193283 | 34.21 | *E. oharae* | 68 |
| RDB15 | 2021 | Paris (RDB) | Vaginal swab | ESBL | 191 | 158812 | 36.31 | *E. hoffmannii* | 104 |
| RDB11 | 2023 | Paris (RDB) | Vaginal swab | WT | 131 | 241823 | 47.60 | *E. steigerwaltii* | 106 |
| RDB1 | 2023 | Paris (RDB) | Vaginal swab | ESBL | 180 | 132684 | 38.41 | *E. xiangfangensis* | 182 |
| RDB6 | 2023 | Paris (RDB) | Vaginal swab | ESBL | 90 | 295146 | 43.07 | *E. xiangfangensis* | 344 |
| RDB3 | 2023 | Paris (RDB) | Vaginal swab | WT | 228 | 120382 | 33.78 | *E. steigerwaltii* | 831 |
| RDB4 | 2014 | Paris (RDB) | Vaginal swab | ESBL | 119 | 207050 | 45.73 | *E. xiangfangensis* | UN |
| MTZ S | 2023 | Metz | Stools | HCASE | 153 | 124491 | 25.44 | *E. hoffmannii* | 104 |
| POI1 | NA | Poitiers | Stools | HCASE | 173 | 166870 | 42.74 | *E. oharae* | 108 |
| ACTIV1 | 2022 | Vincennes | Stools | WT | 188 | 117262 | 33.62 | *E. hoffmannii* | 158 |
| ACTIV3 | 2023 | NA | Stools | WT | 194 | 124182 | 38.21 | *E. hoffmannii* | 158 |
| ACTIV4 | 2023 | NA | Stools | WT | 114 | 288695 | 31.43 | *E. steigerwaltii* | 106 |
| ALE | 2017 | Ales | Stools | CPE | 118 | 251070 | 32.18 | *E. steigerwaltii* | 116 |
| AVC | 2023 | Paris (AVC) | Urines | WT | 106 | 298416 | 26.39 | *E. steigerwaltii* | 45 |
| LIM | 2023 | Limoges | Urines | HCASE | 162 | 189041 | 26.61 | *E. steigerwaltii* | 45 |
| NIM | 2023 | Nîmes | Urines | HCASE | 239 | 138233 | 27.73 | *E. steigerwaltii* | 50 |
| MTZ U | 2024 | Metz | Urines | WT | 161 | 160562 | 40.80 | *E. hoffmannii* | 78 |
| RDB16 | 2016 | Paris (RDB) | Urines | ESBL | 265 | 141183 | 28.62 | *E. oharae* | 108 |
| IMM2 | 2024 | Paris (IMM) | Urines | WT | 129 | 259449 | 26.18 | *E. steigerwaltii* | 113 |
| POI2 | NA | Poitiers | Urines | ESBL | 214 | 225705 | 30.91 | *E. xiangfangensis* | 114 |
| RDB12 | 2014 | Paris (RDB) | Urines | ESBL | 169 | 196351 | 33.14 | *E. xiangfangensis* | 136 |
| RDB13 | 2015 | Paris (RDB) | Urines | ESBL | 213 | 153607 | 33.33 | *E. xiangfangensis* | 144 |
| RDB9 | 2024 | Paris (RDB) | Urines | WT | 198 | 86157 | 30.45 | *E. hoffmannii* | 310 |
| BES | 2023 | Besançon | Urines | WT | 193 | 131607 | 32.64 | *E. hormaechei* | 528 |
| CLE1 | 2022 | Rouen | Protected distal aspiration | WT | 239 | 163725 | 30.71 | *E. steigerwaltii* | 134 |
| CLE2 | 2022 | Moulins | Urines | WT | 207 | 153968 | 33.25 | *E. steigerwaltii* | 50 |
| REN | NA | Rennes | NA | ESBL | 245 | 93833 | 34.02 | *E. hoffmannii* | 97 |
| HEM2 | 2022 | Paris (RDB) | Stools | CPE + ESBL | 247 | 103465 | 40.84 | *E. hoffmannii* | 97 |
| HEM3 | 2022 | Paris (RDB) | Stools | CPE + ESBL | 253 | 102480 | 45.96 | *E. hoffmannii* | 97 |
| HEM4 | 2022 | Paris (RDB) | Stools | CPE + ESBL | 262 | 85144 | 39.83 | *E. hoffmannii* | 97 |
| HEM5 | 2023 | Paris (RDB) | Stools | CPE + ESBL | 204 | 113717 | 36.92 | *E. hoffmannii* | 97 |
| TRS1 | 2023 | Paris (TRS) | Stools | ESBL | 321 | 148456 | 36.97 | *E. hoffmannii* | 168 |
| TRS2 | 2023 | Paris (TRS) | Stools | CPE | 219 | 158854 | 46.09 | *E. steigerwaltii* | 88 |
| TRS3 | 2023 | Paris (TRS) | Stools | ESBL | 358 | 130277 | 35.35 | *E. hoffmannii* | 168 |
| TRS4 | 2023 | Paris (TRS) | Stools | CPE + ESBL | 310 | 130277 | 28.53 | *E. hoffmannii* | 168 |
| TRS5 | 2023 | Paris (TRS) | Stools | ESBL | 308 | 125389 | 29.48 | *E. hoffmannii* | 168 |
| TRS6 | 2023 | Paris (TRS) | Tracheal aspiration | ESBL | 299 | 148456 | 31.05 | *E. hoffmannii* | 168 |
| TRS7 | 2023 | Paris (TRS) | Stools | ESBL | 337 | 98248 | 41.78 | *E. hoffmannii* | 135 |
| TRS8 | 2023 | Paris (TRS) | Stools | ESBL | 197 | 230848 | 35.37 | *E. steigerwaltii* | 110 |
| TRS9 | 2023 | Paris (TRS) | Stools | ESBL | 341 | 148456 | 37.46 | *E. hoffmannii* | 168 |
| NNA1 | 2024 | Paris (RDB) | Stools | ESBL | 205 | 204814 | 36.54 | *E. steigerwaltii* | 124 |
| NNA2 | 2023 | Paris (RDB) | Stools | ESBL | 209 | 132564 | 40.62 | *E. xiangfangensis* | 182 |
| NNA3 | 2023 | Paris (RDB) | Stools | ESBL | 208 | 155592 | 29.53 | *E. xiangfangensis* | 182 |
| NNA4 | 2024 | Paris (RDB) | Stools | ESBL | 227 | 166442 | 31.04 | *E. steigerwaltii* | 124 |
| NNA5 | 2024 | Paris (RDB) | Stools | ESBL | 193 | 218243 | 42.88 | *E. steigerwaltii* | 124 |

NA, not available; ESBL, extended-spectrum beta-lactamase; CPE, carbapenemase-producing Enterobacterales; HCASE, cephalosporinase hyperproduction; WT, wild type; AVC, Avicennes Hospital; FSEF, Fondation Santé des Etudiants de France; IMM, Institut Mutualiste Montsouris; LMR, Louis Mourier Hospital; RDB, Robert Debré Hospital; SLS, Saint Louis Hospital; TRS, Armand Trousseau Hospital
